# Expansins: roles in plant growth and potential applications in crop improvement

**DOI:** 10.1007/s00299-016-1948-4

**Published:** 2016-02-18

**Authors:** Prince Marowa, Anming Ding, Yingzhen Kong

**Affiliations:** Key Laboratory for Tobacco Gene Resources, Tobacco Research Institute, Chinese Academy of Agricultural Sciences, Qingdao, 266101 People’s Republic of China

**Keywords:** Cell wall, Expansin, Plant growth, Cell wall loosening, Crop improvement, Abiotic stress, Biotic stress

## Abstract

*****Key message***:**

**Results from various expansin related studies have demonstrated that expansins present an opportunity to improve various crops in many different aspects ranging from yield and fruit ripening to improved stress tolerance.**

**Abstract:**

The recent advances in expansin studies were reviewed. Besides producing the strength that is needed by the plants, cell walls define cell shape, cell size and cell function. Expansins are cell wall proteins which consist of four sub families; α-expansin, β-expansin, expansin-like A and expansin-like B. These proteins mediate cell wall loosening and they are present in all plants and in some microbial organisms and other organisms like snails. Decades after their initial discovery in cucumber, it is now clear that these small proteins have diverse biological roles in plants. Through their ability to enable the local sliding of wall polymers by reducing adhesion between adjacent wall polysaccharides and the part they play in cell wall remodeling after cytokinesis, it is now clear that expansins are required in almost all plant physiological development aspects from germination to fruiting. This is shown by the various reports from different studies using various molecular biology approaches such as gene achieve these many roles through their non-enzymatic wall loosening ability. This paper reviews and summarizes some of the reported functions of expansins and outlines the potential uses of expansins in crop improvement programs.

## Introduction

When the expansins were first discovered in cucumber hypocotyls (McQueen-Mason et al. 1992), they were reported to loosen plant cell walls in a non-enzymatic but pH dependent manner. The plant cell wall which consists of a primary and secondary cell wall is an important plant feature. Cells of higher plants have a protective cell wall which is basically made up of polysaccharides such as cellulose, hemicellulose and some pectins which are cross-linked together and embedded in an amorphous gel-like matrix. The molecular lengths of some hemicellulosic polysaccharides like xyloglucans are longer than the distance between cellulose microfibrils. This enables them to cross-link adjacent cellulose microfibrils to tether together and coat the surface of the cellulose microfibrils thus forming the cellulose/hemicellulose network that functions as the major tension-bearing framework of the primary cell wall and conferring extensibility to the network structure (Fukuda 2014). The dynamics of the cell wall determines cell shape, functions during development, responde to environmental cues and contributing to the strength and structural integrity of the cell and the whole plant at large.

There is constant assembly, remodeling and disassembly of the cell wall during the plant’s lifetime. This is achieved through the action of the many various types of structural and functional components such as expansins which are secreted into the cell wall space (Fukuda 2014). This constant assembly, remodeling and disassembly of the cell wall is necessary for plant growth and acclimatization. Fukuda (2014) defined cell wall loosening as a continuous reduction in cell wall tensile strength and highlighted that this cell wall loosening is a direct cause of cell wall expansion which subsequently results in cell expansion. This process is crucial because it is the basis of plant growth. The rearrangement of the cellulose/xyloglucan networks which is thought to be achieved through either the remodeling action of expansin genes or molecular grafting between xyloglucan cross-links by means of endotransglucosylation reaction (Fukuda 2014) is vital for plant growth and development.

The cell wall plays crucial roles in various cell activities such as differentiation, transport and communication, senescence, abscission, plant-pathogen interactions and ultimately plant growth. It provides both the mechanical strength needed by the plant and the plasticity that is necessary for the development of plant tissues and organs. Since plant growth can be generalized as a function of cell size and cell number, plant growth and development therefore requires modulation of cell size and shape, which is accomplished by regulated changes in cell wall plasticity. This makes expansins very important since they are actively involved in this area (Cosgrove 2000, 2015; Fukuda 2014; McQueen-Mason et al. 1992; Sampedro and Cosgrove 2005; Zou et al. 2015). Although the expansin’s biochemical working mechanism is not completely understood, it is generally agreed that the action of expansin on the cell wall brings about this much needed plasticity (Cosgrove 2000). Biomechanical analysis by creep tests showed that *AtEXLA2* overexpression has the ability to decrease the wall strength in *Arabidopsis thaliana* (*Arabidopsis*) hypocotyls (Boron et al. 2015).

Expansins comprise a large gene super-family which codes for small (225–300 amino acid residues) cell wall proteins (Fukuda 2014; Sampedro and Cosgrove 2005). According to Kende et al. (2004) they can be divided into four sub families; α-expansin or expansin A (hereinafter referred to as “EXPA”), β-expansin or expansin B (hereinafter referred to as “EXPB”), expansin-like A (hereinafter referred to as “EXPLA”) and expansin-like B (hereinafter referred to as “EXPLB”). Choi et al. (2008) concurred with this classification but went on to add expansin-like X (hereinafter referred to as “EXLX”) as another group of expansins which are remotely related to expansin genes and found both inside and outside the plant kingdom. The classification of expansin and expansin-like genes is based on their phylogenetic relationship and this has been extensively reviewed (Kende et al. 2004; Lee et al. 2001; Li et al. 2003b).

Expansins have the ability to non-enzymatically trigger a pH dependent relaxation of the cell wall which loosens and softens it thus enabling cell expansion. It has been noted that due to the action of expansins, growing plant cell walls extend faster at low pH (4.5), a phenomenon which Rayle and Cleland (1992) preferred to call acid growth. This pH change is brought about by the action of the H^+^ ATPase in the plasma membrane which pumps protons into the cell wall (Cosgrove 2000). However, besides pH, the action of expansins can also be influenced by several other factors including environmental factors (Brummell et al. 1999) such as flooding (Vreeburg et al. 2005) or submergence (Lee and Kende 2001) and hormones like abscisic acid, indole-3-acetic acid (Zhao et al. 2012), auxins (McQueen-Mason et al. 1992), brassinosteroids (Park et al. 2010), cytokinins (Downes and Crowell 1998) and ethylene (Belfield et al. 2005). In this review we will not dwell much on the history, classification and structure of expansins since these and other related matters have been extensively dealt with in earlier reviews (Choi et al. 2008; Cosgrove 2015; Cosgrove et al. 2002; Lee et al. 2001; Sampedro and Cosgrove 2005). This paper will focus mainly on the recent progress and findings from expansin related research and highlights possible uses of expansins in crop improvement programs since an earlier review by Choi et al. (2008) covered a lot of ground with respect to nomenclature of expansin genes, their evolution, biochemical and biophysical properties and their relationship with plant growth and development.

As stated earlier on, plant growth results from an increase in cell size and cell number, thus making cell expansion an important aspect of plant growth and development. This cell expansion however must overcome resistance from the protective cell wall. Among other possible means, cell expansion is achieved through the action of expansin genes on the cell wall where they are thought to act like a zipper and break the hydrogen bonds linking cell wall polysaccharides (Bashline et al. 2014). Although the details of expansin action have not yet been fully elucidated (Dal Santo et al. 2013) they are reported to target hydrogen bonds linking cellulose and hemicellulose especially xyloglucan thus loosening the cell wall. This enables the cell wall polymers to slide and consequently allowing the cell to expand (Bashline et al. 2014; Fukuda 2014).

**Fig. 1:**
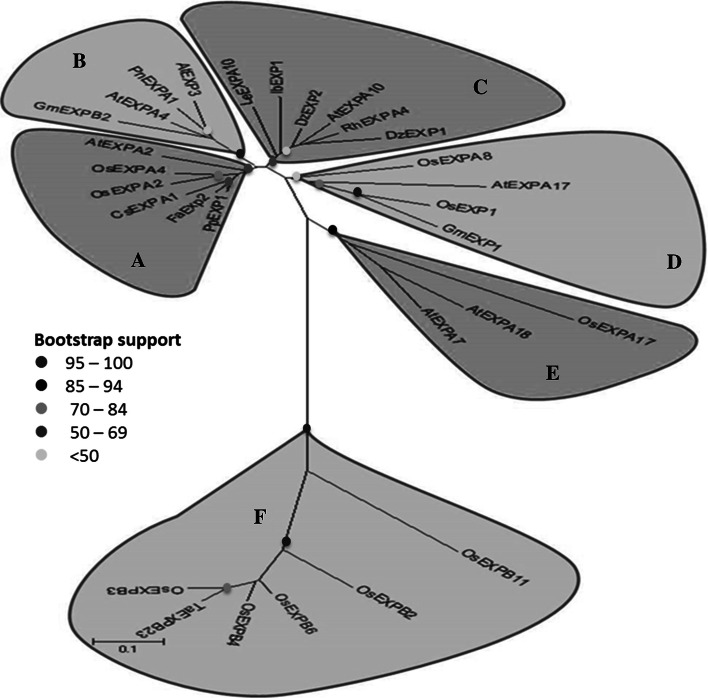
Evolutionary relationship of 29 selected expansin genes and their effect on plant growth. These genes were selected based on the fact that they are phylogenetically and functionally related. Clades *D* and *E* consist of expansins which have been shown to or are thought to act on internodes and roots respectively while clade *F* consists of expansins affecting either root or internode development. Clade *A* on the other hand consists mainly of those expansins affecting germination but it also contains other expansins affecting leaf development. Most of the genes in Clade *B* enhanced overall plant growth when overexpressed while Clade *C* consists of expansins affecting mainly leaf growth, seed germination and fruit ripening. The evolutionary history was inferred using the neighbor-joining method with 500 bootstrap replicates. The optimal tree with the sum of branch length = 5.85540671 is shown. The evolutionary distances were computed using the Poisson correction method and are in the units of the number of amino acid substitutions per site. Evolutionary analyses were conducted in MEGA6

Results from many experiments have shown that expansins are very important to plants. It has been demonstrated that expansins affect almost all plant growth phases and have the potential to influence plant-biotic/abiotic stress relationship (Table 1). Phylogenetic analysis of some of the studied expansin genes shows that different expansins from various species falling within the same clade have almost similar effects on plant growth and development (Fig. 1). Although expansin studies have covered all the sub families, most of the studies have however focused a lot on the expansin A and B sub-families. Clades D and E in Fig. 1 consist of expansins from which have been shown to or are thought to act on internodes and roots, respectively while clade F mainly consists of expansins affecting either root or internode development. Clade A on the other hand consists mainly of those expansins which affect germination but it also contains other expansins affecting leaf development. Despite Clade B and C being mixed bags, it is clear that most of the genes in Clade B resulted in enhanced overall plant growth when overexpressed while Clade C consists of expansins affecting mainly leaf growth, seed germination and fruit ripening. This information is vital especially for future studies and crop improvement programs.

**Table 1:** Selected examples of studies reporting the effects of expansins on plant development and stress adaptation

Expansin name	Sub-family	Mode of expression	Observed phenotype	References
*AtEXPA1*	α-Expansin	Overexpression and inhibition	Increased rate of light-induced stomatal opening and reduced sensitivity of stomata to the stimuli, respectively	Wei et al. (2011a, b)
*AtEXPA2*	α-Expansin	Overexpression and suppression	Overexpressors germinated faster than wild type plants while germination was delayed in mutant lines	Yan et al. (2014)
*AtEXP3*	α-Expansin	Overexpression	Enhanced growth and larger leaves under normal growth conditions	Kwon et al. (2008)
*AtEXPA4*	α-Expansin	Expression profile analyses	Thought to soften the cell wall of the stigma	Mollet et al. (2013)
*AtEXPA7*	α-Expansin	Overexpression	Influenced root hair initiation and root growth	Cho and Cosgrove (2002)
*AtEXPA10*	α-Expansin	Overexpression	Large plant cells, larger leaves and longer stems	Kuluev et al. (2012)
*AtEXPA17*	α-Expansin	Overexpression and knock down	Enhanced and reduced lateral root formation, respectively	Lee and Kim (2013)
*AtEXPA18*	α-Expansin	Overexpression	Influenced root hair initiation and root growth	Cho and Cosgrove (2002)
*LeEXPA1*	α-Expansin	Expression analysis	Proposed to be involved in fruit softening	Rose et al. (1997, 2000)
*LeEXP1*	α-Expansin	Overexpression and Suppression	Overexpression of the gene resulted in softer fruits while its suppression produced firmer fruits in transgenic tomatoes	Brummell et al. (1999)
*LeEXPA8*	α-Expansin	mRNA expression analysis	Thought to influence germination since it is expressed in germinating seeds only and appears to be involved during the initial elongation of the radicle	Chen et al. (2001)
*LeEXPA10*	α-Expansin	mRNA expression analysis	Thought to influence germination as well as seed development	Chen et al. (2001)
*SlExp1*		Knockout	Increased fruit firmness	Minoia et al. (2015)
*OsEXPA1*	α-Expansin	Expression analysis	Thought to influence coleoptile and internode development	Cho and Kende (1997b)
*OsEXPA4*	α-Expansin	Overexpression Antisense (RNAi)	Pleiotropic phenotypes in plant height, leaf number, flowering time and seed set as well as enhanced coleoptile growth Shorter plants, decreased coleoptile and mesocotyl lengths	Choi et al. (2003) Zou et al. (2015)
*OsEXPA8*	α-Expansin	Overexpression	Increased root mass, number and size of leaves as well as plant height	Ma et al. (2013)
*OsEXPA17*	α-Expansin	Overexpression	Influenced rice root development	Yu et al. (2011)
*DzEXP1*	α-Expansin	Expression analysis	Thought to be involved in fruit/pulp softening and peel dehiscence	Palapol et al. (2015)
*NtEXPA5*	α-Expansin	Overexpression	Increased organ size especially the leaves and the stem	Kuluev et al. (2013)
*DzEXP2*	α-Expansin	Expression analysis	Thought to be involved in fruit/pulp softening as well as peel dehiscence	Palapol et al. 2015)
*FaExp2*	α-Expansin	Expression analysis	Thought to take part in cell wall polymer disassembly during fruit ripening	Civello et al. (1999)
*MaExp1*		Overexpression	Thought to affect banana ripening	Asif et al. (2014)
*PpEXP1*	α-Expansin	Overexpression	Enhanced germination and abiotic stresses tolerance	Xu et al. (2014)
*RhEXPA4*	α-Expansin	Overexpression Overexpression and silencing	Higher germination percentage; increased lateral root formation and modified leaves Affected expansion and dehydration tolerance of rose petals	Lü et al. (2013) Dai et al. (2012)
*GmEXP1*	α-Expansin	Overexpression	Accelerated root growth	Lee et al. (2003)
*GbEXPATR*	α-Expansin	Overexpression	Enhanced root hair development in transgenic Arabidopsis	Li et al. (2015b)
*IbEXP1*		Overexpression	More rosette leaves	Bae et al. (2014)
*PnEXPA1*	α-Expansin	Overexpression	Large plant cells, larger leaves and longer stems	Kuluev et al. (2012)
*CsEXPA1*	α-Expansin	Overexpression	Initiated development of the leaf primordium	Pien et al. (2001)
*AtEXPB1*	β-Expansin	Overexpression	significantly longer petioles under normal growth conditions	Kwon et al. (2008)
*AtEXPB5*	β-Expansin	Expression profile analyses	Thought to soften the cell wall of the stigma	Mollet et al. (2013)
*OsEXPB2*	β-Expansin	Expression analysis Silenced	Thought to influence root hair and internodes development Confirmed the earlier suggested role as shown by physiological changes including reduced root and leaf sizes	Cho and Kende (1997b) Zou et al. (2015)
*OsEXPB3*	β-Expansin	Expression analysis	Thought to be involved in internode elongation as well as root development	Cho and Kende (1997b; Lee and Kende (2001)
*OsEXPB4*	β-Expansin	Expression analysis	mRNA accumulation correlated well with internode elongation	Lee and Kende (2001)
*OsEXPB6*	β-Expansin	Expression analysis	mRNA accumulation correlated well with internode elongation	Lee and Kende (2001)
*OsEXPB11*	β-Expansin	Expression analysis	mRNA accumulation correlated well with internode elongation	Lee and Kende (2001)
*GmEXPB2*	β-Expansin	Overexpression	Enhanced overall plant growth, higher root cell division and elongation. Enhanced phosphorus uptake	Guo et al. (2011)
*GmEXPB2*	β-Expansin	Overexpression	Increase in phosphorus efficiency	Zhou et al. (2014)
*TaEXPB23*	β-Expansin	Overexpression	Improved tolerance of transgenic tobacco plants to oxidative stress Overexpressors performed better under drought. They showed enhanced root growth and water stress tolerance	Han et al. (2015) Li et al. (2015a)
*TaEXPB23*	β-Expansin	Overexpression	Longer internodes, larger leaf blades, more leaves, more roots	Xing et al. (2009)
*HvEXPB1*	β-Expansin	Promoter deletion	Shown to influence root hair formation	Won et al. (2010)
*AtEXLA2*	Expansin-like A	Overexpression	Longer roots which were significantly longer than the wild type roots	Boron et al. (2015)
*AtEXPLA2*	Expansin-like A	Overexpression and mutant lines	Reduced *EXLA2* transcript levels enhanced resistance to necrotrophic pathogens (*Botrytis cinerea; Alternaria brassicicola*)	Abuqamar et al. (2013)

The table shows expansin genes from several species affecting various stages of plant development from seed germination to fruiting as well as those affecting plant’s response to environmental cues. It also shows the effects of manipulating expansin genes on plant growth using various molecular biology tools

*Pp Prunus persica*, *Le/Sl Lycopersicon esculentum/Solanum lycopersicum L.*, *At Arabidopsis thaliana*, *Ta Triticum aestivum*, *Gb* Gossypium barbadense, *Rh Rosa hybrid*, *Gm Glycine max*, *Os Oryza sativa*, *Ib Ipomoea batatas*, *Cs Cucumis sativus*, *Pn Populus nigra*, *Dz Durio zibethinus*, *Fa Fragaria x ananassa*, *Ma Musa acuminate*

Besides affecting particular growth stages, expansins have also been shown to play a pivotal role in enhancing plant’s ability to withstand biotic and abiotic stresses (Li et al. 2011, 2013; Xu et al. 2014; Yan et al. 2014; Zörb et al. 2015).

## Effects of expansins on specific plant development stages from germination to fruiting

### Effects on germination

Germination is a process which is regulated by hormones such as ABA and GA which induces and breaks dormancy, respectively (Holdsworth and Soppe 2008). In general terms, it begins with imbibition which leads to the cracking of the seed coat thus allowing the radicle to emerge (Finch-savage and Leubner-metzger 2006). The various aspects of seed dormancy and germination and related molecular networks have been extensively reviewed (Finch-savage and Leubner-metzger 2006; Holdsworth and Soppe 2008). For farmers, it is usually encouraged to use seeds with a higher germination percentage which will encourage uniform seedling emergence since this will have in most cases a direct bearing on costs and yield among other parameters.

During the early phases of germination, the seed undergoes a lot of transcriptional changes of key metabolic enzymes and several expansins are induced (Weitbrecht et al. 2011). Some of these expansins such as the tomato expansin *LeEXPA8* for example, are only expressed in germinating seeds (Chen et al. 2001). Expansins such as *AtEXPA1*, -*2*, -*8*, and -*9* (Morris et al. 2011; Weitbrecht et al. 2011; Yan et al. 2014), *LeEXPA8* and *LeEXPA10* (Chen et al. 2001) and *OsEXPA1* and *OsEXPA2* (Huang et al. 2000) are also believed to play important roles during seed germination. Messenger RNA expression analysis have demonstrated that seed germination coincides with strong expression of these expansins thus supporting the hypothesis that expansins play important roles in endosperm-mediated processes during early germination that lead to and control testa rupture. This notion has been supported by Lü et al. (2013) who reported that after overexpressing *RhEXPA4*, a rose expansin gene in *Arabidopsis*, the germination percentage of transgenic Arabidopsis seeds was higher than that of wild type seeds even under salt stress and ABA treatments. This was further supported by Yan et al. (2014) who also demonstrated that overexpression of *AtEXPA2* hastened germination while its suppression significantly delayed it. Yan et al. (2014) also demonstrated that *AtEXP2* is likely to control seed germination through GA signaling.

Although the details of expansin action on germination are not yet clear, the need for cell wall loosening and the involvement of expansins during germination have been endorsed by Voegele et al. (2011) who suggested that *AtEXPA9* is involved in micropylar endosperm weakening and in radicle growth in *Arabidopsis*. These researchers confirmed the importance of cell wall loosening in many plant developmental stages including seed germination where radicle growth and endosperm weakening take place (Voegele et al. 2011). In a separate study, this *AtEXPA9* was also reported to be involved in seed germination together with *AtEXPA2* and *AtEXPA7* (Morris et al. 2011). Recent studies have also reported that *atexpb2* mutant plants showed a significantly lower germination rate than the wild type plants under different levels of Methyl viologen (oxidative stress) treatment thus suggesting that expansin proteins are involved in oxidative stress tolerance as well (Han et al. 2015).

With this information at hand, one can easily conclude that expansins are indeed important during seed germination. Combined with other methods, such expansins can be a useful tool in enhancing this process and improving the ability of the seed to germinate under various conditions. Such conditions include salinity which is fast becoming a common problem affecting crop production.

### Effects on root development and growth

Besides anchorage, plant roots play other crucial roles which include nutrient and water uptake thus a vigorous root system is needed since it will generally result in a better plant. The expression of the β-expansin gene *HvEXPB1*was demonstrated to be root hair-specific and associated with root hair formation in barley (Kwasniewski and Szarejko 2006; Won et al. 2010). *HvEXPB1* gene contains five root hair-specific cis-elements (RHEs) in its promoter region (Kwasniewski and Szarejko 2006) and it has been confirmed that these RHEs play vital roles in cell wall modification during root hair morphogenesis (Won et al. 2010).

Several other expansins have also been shown to influence root development and growth. These include *GmEXP1*, a root specific α-expansin (Lee et al. 2003) which was found to accelerate root growth when ectopically expressed (Guo et al. 2011). Besides enhancing the overall plant growth when overexpressed, *GmEXPB2*, another expansin gene from soybean, was also found to be involved in root hair elongation (Guo et al. 2011). Using quantitative real-time PCR technique to evaluate the temporal and spatial expression patterns of *GmEXPB2*, Guo et al. (2011) found out that its expression occurred primarily in the roots and was up-regulated by abiotic stresses including water, phosphate (Pi) and iron (Fe) deficiency. It was also reported that overexpression of *RhEXPA4*, a rose expansin gene resulted in transgenic Arabidopsis plants with more lateral roots (Lü et al. 2013). An almost similar report showed that transgenic plants overexpressing *TaEXPB23* exhibited higher fresh weight and longer primary root than wild types under oxidative stress (Han et al. 2015).

Through loss-of-function studies using the RNA interference approach together with gain-of-function studies, several other expansins have been shown to promote and facilitate root initiation, root hair initiation and lateral root emergence in plants. Compared to their wild type counterparts, plants with knocked down *AtEXPA17* exhibited reduced lateral root formation while overexpressors of the same gene showed enhanced lateral root formation in transgenic Arabidopsis (Lee and Kim 2013). The same trend was reported in rice when *OsEXPB2* was silenced (Zou et al. 2015). Silencing *OsEXPB2* was shown to affect root system architecture by inhibiting cell growth. Many other expansin genes have also proved to play important roles during root development and growth. Such expansins include *TaEXPB23* which has been shown to significantly increase root network and root biomass when overexpressed (Li et al. 2015a; Xing et al. 2009), *OsEXPA17* which has been reported to influence rice root development (Yu et al. 2011), *AtEXPA7* and *AtEXPA18* which have been shown to play crucial roles during root hair initiation and root growth in Arabidopsis (Cho and Cosgrove 2002) and *AtEXP4* (Lee and Kim 2013). Recent studies have also shown that overexpression of *AtEXLA2*, a member of the expansin-like sub-family resulted in roots which were significantly longer than those of the wild type (Boron et al. 2015).

Although the clear mode of action of expansin on root development and growth is not clear as yet, it has been demonstrated that expansins accelerate cell growth and expansion when exogenously applied to isolated living cells (Cho and Cosgrove 2000). It is also known that expansins increase the cell wall flexibility through loosening and softening cell walls thus enabling necessary tissue modification. This and other related knowledge can be put together and included in crop improvement programs. Although most desired crop traits are controlled by many genes, inclusion of certain expansins in the breeding programs can produce beneficial effects. It has been shown that overexpression of *GmEXPB2* enhances root growth under stress conditions (Guo et al. 2011) thus presenting a possibilty to improve crops for specific regions. Besides enhancing rice and soybean root systems, GmEXPB2 and OsEXPB2 have been reported to enhance the plants’ ability to tolerate abiotic stresses. If such expansins are employed in breeding programs, it is likely that these crops will be significantly improved.

### Effects on leaf initiation and leaf growth

Leaves act as the manufacturing industries for the plant and animals. The process of leaf development is complex and influenced by many factors including hormones and genes such as expansins. According to Green (1999), a new leaf emerges in the region of reduced tension, which he reported to be surrounded by a circular zone of elevated tension. He pointed out that the tissue tension depends on mechanical properties of cell walls and therefore among other things, we can assume the involvement of expansins since they have been reported to influence the mechanical properties of the cell walls. Green (1999) further pointed out that the leaf primordium is initiated in the site of the peripheral region of apical dome where the cell wall extensibility is elevated.

This elevated extensibility of the cell wall could be a result of expansin action. A follow up study by Pien et al. (2001) confirmed that indeed expansins are involved during leaf initiation. They reported that when *CsEXPA1* expression was ectopically induced, it initiated development of the leaf primordium, which later developed into a normal leaf. In their study with tetracycline-induced expansin expression, they also found that local application of the tetracycline-containing paste at early stages of new primordium development was effective for the induction of a lobe on a leaf blade thus supporting the hypothesis that expansin play an important role during leaf development.

Several later studies have confirmed and demonstrated that expansins play an important role during leaf initiation and growth. Using a special method that allowed transient local micro-induction of gene expression in transgenic plants, Pien et al. (2001) tested the possible function of expansins in leaf morphogenesis. Results from their study confirmed the earlier notions as it showed that local expression of expansins within the meristem induces a developmental program that recapitulates the entire process of leaf formation. Many other studies have also demonstrated that overexpression or suppression of expansin genes can positively or negatively affect the process of leaf development, respectively. Using quantitative real-time PCR technique to evaluate the expression patterns of *AtEXP10*, Cho and Cosgrove (2000) demonstrated that there was much greater *AtEXP10* expression in young growing petioles and leaf blades than in older non-growing leaves which highlights the significance of expansin genes during the process of leaf initiation and development. Overexpression and suppression studies of this expansin gene revealed that leaf size was substantially reduced in antisense lines while its overexpression resulted in plants with somewhat larger leaves (Cho and Cosgrove 2000). Also, *AtEXPA10* and *PnEXPA1* were reported to significantly affect tobacco leaf cell sizes resulting in larger leaves when overexpressed (Kuluev et al. 2012, 2013). This clearly supports the idea that *AtEXP10* functions in the control of leaf size through its action on cell-wall rheology.

Recent studies have concluded that expansins are involved in wheat leaf growth. Zhou et al. (2015) reported that expansin activity was associated with the relative elongation rate of leaves during leaf development. Other related examples include the suppression of *OsEXPB2* in rice which resulted in significant physiological changes including a significant reduction in the width of leaf blades (Zou et al. 2015) and overexpression of *IbEXP1* which resulted in transgenic plants with more rosette leaves (Bae et al. 2014).

Also, abiotic factors like vapour pressure deficit can negatively affect the expansion of the leaf through their effect on expansin expression. They have been reported to down-regulate the transcript level of expansin genes thus affecting cell extensibility which consequently reduces leaf growth and development (Devi et al. 2015). All this demonstrates the involvement of expansins in leaf development thus we can speculate that combined with other breeding strategies, these expansins presents an opportunity for scientists to improve fodder and other crops whose leaves are of economic importance. This can also see yield increase due to enhanced light interception which will allow for increased photosynthesis.

### Stomata opening and closing

Features such as trichomes and stomata play vital roles in enabling plants to adopt and thrive in their environments. The opening and closing of stomata is strictly regulated by various intracellular and extracellular factors in response to environmental cues (Wei et al. 2011a, b). Guard cell expressed expansins, *AtEXPA1* and *VfEXPA1* regulate stomatal opening by altering the structure of the guard cell wall (Wei et al. 2011a, b). Overexpression of these expansins in Arabidopsis and tobacco plants respectively increased the rate of light-induced stomatal opening, while their inhibition reduced the sensitivity of stomata to the same stimuli (Wei et al. 2011a, b). These researchers also reported an increase in transpiration and photosynthesis rate in overexpressors of these genes which was almost double that of the wild type plants. This led them into concluding that expansins participate in the regulation of stomatal movement by modifying the cell walls of guard cells basing on the fact that wall loosening, which is controlled by expansins, is essential for guard cell expansion and constriction.

It has also been noted that under drought conditions, *RhEXPA4* overexpression has the potential to enhance the survival rate of the transgenic plants (Lü et al. 2013). Lü et al. (2013) concluded that among other probable causes, enhanced drought tolerance of *35S::RhEXPA4* plants was partially a result of decreased stomatal density due to leaf modifications in *RhEXPA4* overexpressors. These overexpressors developed smaller rosette size with compact epidermal cells, indicating that *RhEXPA4* improves drought tolerance by modulating leaf growth. All these recent findings are in line with Sampedro and Cosgrove (2005) who hypothesized that the overexpression of expansin might disrupt the elaborate microtubule arrays, cellulose deposition and cell-wall thickening that are required for the development of stomatal guard cells and their adjacent cells during stomatal morphogenesis thus resulting in altered leaf morphology.

One strategy through which plants acclimatize or adapt to drought is through reducing transpirational water loss. Stomatal closure or lower stomatal density has been mentioned among the earliest responses to drought stress (Chaves et al. 2003). Combined with other tools, expansins such as *RhEXPA4* and *AtEXP1* can be useful crop improvement tools in this regard considering the current challenges that crop production is facing.

### Effects on stem elongation

After emerging from the soil, most of the plants must have a mechanism that enables them to increase their stem sizes before they can start bearing fruits. Although wheat breeding programs have favored shorter varieties because of their ability to withstand lodging, the need for longer stems or the ability to develop them when the need arises might be very important in crops like rice which are usually grown on lowlands and thus prone to flooding.

Catling et al. (1988) outlined that although rice has a good reputation for growing well under flooded conditions, it should however possess the ability to escape aerobically from rising water to maintain the apical parts above water. This is very important or else the results will be fatal. Some rice varieties have the ability to accelerate stem elongation in response to flooding environment. After reports of unbelievable rice stem elongation rates of up to 25 cm per day (Lee and Kende 2002), several researcher focused their attention on rice stem elongation. Most of them reported the involvement of expansins (Cho and Kende 1997a, b; Choi et al. 2003; Zou et al. 2015).

Following a report by Cho and Kende (1997b) which stated that the expression of *OsEXPA2* and *OsEXPA4* was induced by submergence and treatment with gibberellin, crop scientists wanted to learn more about the involvement of expansins in rice stem elongation. This saw several expansin genes including *OsEXPB3*, *OsEXPB4*, *OsEXPB6*, and *OsEXPB11* being implicated in rice stem elongation (Lee and Kende 2001). Although the exact mode of action is still not clear, it is generally agreed and hypothesized that expansins break the hydrogen bonds between cellulose microfibers and cross-linking matrix glycans, resulting in slippage between cell walls (Zou et al. 2015). This enables cells to expand while allowing tissues to differentiate and grow accordingly and in this case thus stem elongation.

Choi et al. (2003) concurred with Lee and Kende (2001) and demonstrated that expansins such as *OsEXP4* affects rice stem sizes. Even though overexpression of *OsEXP4* resulted in pleiotropy, their results showed that overexpressors carrying a single copy of the gene grew taller than control plants and developed some additional leaves while the antisense plants were shorter than the average control plants (Choi et al. 2003). This action of expansin is consistent with findings reported by Zou et al. (2015) who noted a decrease in plant height of RNAi lines where expansins were suppressed. Zou et al. (2015) also showed that wild type plants were taller than the RNAi lines by almost 12 cm thus confirming the involvement of expansin in stem development and elongation. Besides affecting stems, expansins also affect coleoptile and mesocotyl lengths. *OsEXP4* overexpressors demonstrated a 31 and 97 % increase in coleoptile and mesocotyl length, respectively while in antisense plants, a 28 and 43 % decrease, respectively was noted (Choi et al. 2003). This change can be attributed to the reported increase in average cell length which increased by up to 58 % in the mesocotyls of lines overexpressing *OsEXP4* while it decreased by 22 % in the antisense transgenic lines (Choi et al. 2003). It was also shown that this change in cell length was due to increased and decreased coleoptile cell wall extensibility of sense and antisense transgenic lines, respectively. Cell wall extensibility of coleoptiles from sense transgenic lines increased by up to 32 % while that of antisense transgenic lines decreased by up to 20 % (Choi et al. 2003). These reports have been recently supported by Boron et al. (2015) who reported that *AtEXLA2* overexpression decreased the wall strength in *Arabidopsis* hypocotyls consequently resulting in transgenic plants with significantly longer hypocotyls than in wild type plants.

In short, it has been demonstrated that several expansins (Table 1) have the potential to alter plant cell wall extensibility and subsequently influence stem growth and development through the hypothesized mode of action. With all this information, crop scientist might consider including expansin in their breeding programs. Wheat breeders for example have been screening for genotypes with longer coleoptile lengths. The coleoptile is essential for successful emergence and early plant vigour (Farhad 2014). Plants with longer coleoptiles can be sown deeper and this allows growers to exploit soil moisture lying below the drying topsoil. Deeper sowing also assists in reducing removal of seeds by birds and rodents and in avoiding phytotoxicity associated with some pre-emergent herbicides (Farhad 2014). Overexpression of *OsEXP4* was shown to significantly increase coleoptile and mesocotyl length by a massive 31 and 97 %, respectively (Choi et al. 2003) as described above. This shows that if properly integrated into breeding programs, expansins can be a useful tool.

### Expansin and reproduction

Several expansins are predominantly expressed in the plant reproductive organs. A number of different α-expansins are expressed during floral elongation, opening and senescence in *Mirabilis jalapa* (Dai et al. 2012; Gookin et al. 2003). It is likely that these expansins affect these reproductive growth phases. Dramatic changes in expansin transcript abundance during the rapid expansion and subsequent senescence of the ephemeral flowers suggests that expansins are involved during this period and are thought to play a pivotal role in influencing flower growth and senescence (Gookin et al. 2003). Dai et al. (2012) demonstrated that silencing and overexpressing *RhEXPA4*, a rose expansin, affected expansion and dehydration tolerance of rose petals. In a similar report, the expression of an α-expansin *CpEXP1* was shown to be directly related to the development of winter sweet flowers (Ma et al. 2012). This expression showed an almost similar pattern with the expression of gladiolus α-expansin *GgEXPA1* (Azeez et al. 2010). Real-time PCR results showed that the transcript level of the *CpEXP1* gene in flower buds gradually increased in the early stages of flower development until a peak was reached before it showed a drastic reduction in the final stages of flower development. This leaves us speculating that manipulation of such expansin genes can benefit agricultural sectors like floriculture and horticulture at large.

On the other hand, pollination is a very important part of the reproduction phase in flowering plants. Although not many studies have been done to elucidate the role played by these expansins during pollination (Lausser et al. 2010), pollen tube development and fertilization, several β-expansins have however been reported in rice pollen grains (Dai et al. 2007) and maize pollen grains (Kapu and Cosgrove, 2010; Li et al. 2003a; Valdivia et al. 2006, 2007, 2009). It is believed that these expansins play important roles which include among other things softening the stigma cell walls. This is thought to facilitate and enable penetration and growth of the pollen tube since it must overcome the resistance on the stigma surface, a problem that any other foreign pollen tube or other intruder must face thus protecting the plant from foreign pollen grains and potential pathogens.

This idea has been supported by some researchers who have speculated that by breaking the cellulose–hemicellulose hydrogen bonds of these reproductive structures, the expansins facilitate this penetration by softening the stigma and underlying cell walls (Mollet et al. 2013). The need for stigma cell wall loosening and softening has led researchers to conclude that there is a possibility of the involvement of expansins during pollen tube development and fertilization (Mollet et al. 2013). This idea has been supported by the discovery of *AtEXPA4* and *AtEXPB5* which are strongly expressed in dry pollen grains, during pollen imbibition and during pollen tube growth. Several other expansins which are expressed on the stigma and ovary of Arabidopsis have also been reported (Mollet et al. 2013).

*ZmEXPB1* is a maize pollen expansin which has also been linked with an in vivo wall-loosening function which facilitates pollen tube penetration into maize silk and growth through them (Valdivia et al. 2007). It was noted that silks continued to elongate for longer periods after pollination in the mutant lines lacking the protein coded by this expansin gene (Valdivia et al. 2006, 2007). Valdivia et al. (2009) showed that emerging pollen tubes from pollen deficient in this β-expansin gene had difficulties entering the silk. Kapu and Cosgrove (2010) also reported on this maize expansin and propounded that such specific silk expansins may facilitate pollen tube growth by loosening the maternal cell walls. They however dismissed the hypothesis that silk growth inhibition was associated with a down-regulation of expansin abundance and/or activity which resulted in rigidification of the silk cell walls. They pointed out that cell wall rigidification may occur by a number of mechanisms including the coupling of feruloyl side chains attached to wall polysaccharides, formation of isodityrosine links, and the strengthening of pectin–calcium networks.

Zhang et al. (2014) found that out of the 88 maize expansin genes (*ZmEXPs*), at least 21 were predominantly expressed in reproductive organs. These authors reported that 16 *ZmEXPs* were predominantly expressed in the tassels while 5 *ZmEXPs* were predominantly expressed in the endosperm suggesting their involvement in endosperm development. If these expansins play an important role during the development of the endosperm, they might be a useful tool worth adopting. The endosperm is an important seed component which occupies a huge part of the seed hence integrating these expansins with other maize breeding tools might increase the yield.

All these studies support the hypothesis that expansins are involved during reproduction. They have been endorsed by recent knowledge which outlines the involvement of expansins during this growth stage and states that expansins solubilize the middle lamella and facilitate cell separation which then aids invasion of the maternal tissues (Georgelis et al. 2015).

### Effects on fruit ripening and softening

Hormones and the environment play a crucial role on the growth and development of plants. Ethylene, a ripening hormone, influences the transcription level of *LeEXPA1*, a tomato expansin and there is a positive correlation between *LeEXPA1* level and tomato fruit softening (Rose et al. 1997). It is thought that through the reported action of expansins on cell wall, this ripening-regulated expansin expression is likely to contribute to cell wall polymer disassembly which results in fruit softening by increasing access of specific cell wall polymers to hydrolase action (Rose and Bennett 1999). The role of expansins on fruit ripening has been recently endorsed by Minoia et al. (2015) who concurred with the idea that the expansins that are highly expressed during tomato fruit ripening contribute to the fruit softening. Minoia et al. (2015) demonstrated that mutations in α-expansin *SlExp1* gene increased fruit firmness. They reported a 41 and 46 % fruit firmness enhancement in *Slexp1*-*6* and *Slexp1*-*7* mutant lines, respectively as compared to the control plants.

Xyloglucan disassembly has been implicated as an early event in fruit softening but enzymatic basis for xyloglucan depolymerization is not well established. However, Rose and Bennett (1999) hypothesized that xyloglucan metabolism may be regulated by substrate accessibility and expansins have been proposed to mediate enzymatic accessibility of this substrate in ripening fruit. This is in line with reported effects of the tomato *Exp1* (Brummell et al. 1999). Since softening of tomato fruit during ripening is accompanied by alterations in both the architecture and physicochemical properties of the cell wall, and in the polymers of which it is composed, overexpression of this expansin gene (tomato *Exp1*) has been shown to hasten the softening process (Brummell et al. 1999). The tomato *Exp1* expression was correlated with fruit cell wall hemicellulose depolymerization and fruit softening, typical of ripe fruit, even in mature green fruit before the commencement of ripening (Brummell et al. 1999). This concurs with the observation that in the mutant lines that did not express *LeEXPA1* expansin gene, the tomato fruits remained green and firm (Rose et al. 2000) and is further supported by the recent reports which states that *Slexp1* mutant lines remained firm for longer periods than the wild type tomato plants (Minoia et al. 2015).

Several ripening related expansins have been reported in strawberries (Civello et al. 1999; Harrison et al. 2001)*. FaEXP2* is one example of such expansin genes which is predominantly expressed in strawberry fruits. Its expression has been shown to increase in ripening strawberry fruits (Civello et al. 1999). This points out at its involvement during the ripening process and this has been further supported by Brummell et al. (1999). In durian fruits (*Durio zibethinus)*, *DzEXP1* and *DzEXP2* expression is also positively correlated with durian fruit softening suggesting that these expansins are involved during durian fruit ripening and have been shown to affect peel dehiscence and softening of the fruit pulp (Palapol et al. 2015).

Recent studies have reported ripening related expansins in *Vasconcellea pubescens* and *Magnolia grandiflora* fruits (Gaete-Eastman et al. 2015; Lovisetto et al. 2015). *VpEXPA2* is an α-expansin which has been implicated in softening of *Vasconcellea pubescens* fruits (Gaete-Eastman et al. 2015) while *MgEXP1* and *MgEXP2* have been implicated in *Magnolia grandiflora* softening with *MgEXP2* reported to show a more ripening-related expression (Lovisetto et al. 2015). *MgEXP2* had low transcripts in young growing tissues peaking in the ripe red sarcotesta (Lovisetto et al. 2015), indicating its possible involvement in tissue softening. Several other expansins affecting ripening have been reported in many other crops including peach (Hayama et al. 2000) and banana (Trivedi and Nath 2004).

Breeding for improved fruit shelf life is still a major target for most horticultural crop including tomatoes. The above studies led to the conclusion that expansins play a significant role during fruit ripening and softening. Considering the current huge post-harvest losses being experienced, this knowledge can be used to develop new alleles in different components of the fruit softening pathways which will likely extend breeder’s tool box to improve tomato shelf life for example. This could be useful in breeding programs in combination with other alleles in the antioxidant or ethylene pathway especially when one considers that many of earlier investigations that focused on the manipulation of the polyamine or anthocyanin pathways had deleterious consequences on fruit quality traits such as flavor, texture and aroma despite their contribution in delaying fruit softening (Lovisetto et al. 2015).

### Effects on crop yield

Bae et al. (2014) summarized a number of studies which showed that seed size was altered when the transcript level of the seed development-related genes were modulated. Such genes include expansins which have been implicated in affecting seed development and seed size (Bae et al. 2014; Kuluev et al. 2012). Seed size is one of the traits that breeders are always trying to improve. When a sweet potato β-expansin gene (*IbEXP1*) was overexpressed in Arabidopsis under the control of the cauliflower mosaic 35S promoter, it enhanced plant growth rate (Bae et al. 2014). Most importantly, overexpression of this gene resulted in plants with thicker siliques and produced seeds which were significantly larger than those from Col-0 plants (Bae et al. 2014). Interestingly, these large seeds accumulated more proteins and starch than their control counterparts. In short, the *IbEXP1* overexpressors produced more inflorescence stems and siliques than control plants which led to a 2.1–2.5 fold increase in total seed yield per plant (Bae et al. 2014). An almost similar trend was observed when *AtEXPA10* and *PnEXPA1* genes were overexpressed in tobacco (Kuluev et al. 2012). Overexpression of these expansin genes in tobacco resulted in the tobacco plants producing larger leaves and larger flowers which weighed more than their wild type counterparts (Kuluev et al. 2012).

Expansins do not only affect seed yield, they also have the ability to affect other types of yield which might not necessarily be grain. For example, in cases where tubers are the harvested yield, expansins have been reported to increase the sizes of these tubers (Noh et al. 2013). Messenger RNA expression analysis suggests that expansins might also influence the growth of the cotton fibers (Shimizu et al. 1997). This notion is supported by the presence of *GhEXP1* (Harmer et al. 2002; Shimizu et al. 1997), *GbEXPA2* and *GbEXPATR* (Li et al. 2015c) expansin genes which are predominantly expressed in the cotton fibers where they are thought to play an important role in cell wall loosening during fiber elongation (Harmer et al. 2002). Recent expression analysis, RNAi and overexpression studies have revealed that indeed cotton α-expansin play a significant role in fiber development (Li et al. 2015c). It has been demonstrated that besides enhancing root hair development in transgenic Arabidopsis, *GbEXPATR* overexpression enhanced cotton fiber length, fineness and strength (Li et al. 2015c).

It can therefore be concluded that even though yield is a quantitative trait, expansins can be a useful tool to manipulate yield of many different crops. However, there are some exceptions, for example overexpression of *RhEXPA4* at high levels in Arabidopsis affected fertility, resulting in a reduced number of inflorescences and flowers which subsequently resulted in an 80 % loss in seed production (Lü et al. 2013). There is therefore a need for further research in this area.

### Effects on biotic and abiotic stress tolerance

Just like any other plant, during desiccation the resurrection plant (*Craterostigma plantagineum*) employs many of the protective mechanisms such as the accumulation of sugars and protective proteins (Hoekstra et al. 2001). However, this plant has demonstrated an outstanding ability to survive extreme cases of desiccation which usually result in the death of most other plants. How does it achieve this?

It has been noted that in resurrection plants, cell wall extensibility increased markedly in the leaves during drying and this coincided with an increase in expansin activity (Jones and McQueen-Mason 2004). These researchers noted that transcript abundance for expansin genes correlated closely with the dehydration and rehydration events in the resurrection plant and they concluded that expansins play a key role in enabling desiccation tolerance in this plant. Several studies have provided evidence that expansins are associated with environmental stress tolerance in plants. This idea has been supported by Zhao et al. (2011) who reported enhanced drought tolerance in wheat varieties overexpressing expansin genes. Li et al. (2011) who was in agreement with this idea pointed out that transgenic tobacco lines overexpressing *TaEXPB23* driven by the constitutive 35S cauliflower mosaic virus (CaMV) promoter lost water more slowly than the wild-type plants under drought stress. It was further supported by (Li et al. 2013) who showed that when the same expansin gene, *TaEXPB23*, was expressed in tobacco plants under the control of the stress-inducible promoter RD29A, the transgenic plants became more tolerant to water stress than their wild type counterparts.

Abiotic stresses, such as drought, cold and salinity result in the production and accumulation of reactive oxygen species (ROS). These are highly reactive and toxic to plant cells. *TaEXPB23* has been reported to influence the activity of antioxidant enzymes: in particular, the activity of the cell wall-bound peroxidase (Han et al. 2015). Han et al. (2015) showed that overexpression of *TaEXPB23* improved the tolerance of transgenic tobacco plants to oxidative stress. This is in line with earlier reports by Abuqamar et al. (2013) who reported an enhanced tolerance to phytoprostance A_1_ in *Atexpla2* mutant lines. Other recent studies have concluded that expansins are involved in wheat response to water stress (Zhou et al. 2015) thus endorsing the idea that expansins play a key role in enabling drought tolerance in plants.

Heat and salt stress are both detrimental abiotic stresses that can cause serious damage to crops. However, expansins can enhance the plant’s ability to withstand such stress as shown by various studies. Overexpressing the α-expansin gene *PpEXP1* from *Poa pratensis* in tobacco plants produced transgenic plants which exhibited a less structural damage under heat stress (Xu et al. 2014). These transgenic plants showed lower electrolyte leakage, lower levels of membrane lipid peroxidation, and lower content of hydrogen peroxide. On the other hand, they also showed a higher chlorophyll content, a higher net photosynthetic rate, a higher relative water content, a higher activity of antioxidant enzyme and a higher seed germination rate compared to the wild-type plants (Xu et al. 2014). This effect of *PpEXP1* is almost similar to the effect of *RhEXPA4* which among many other things conferred abiotic stress tolerance when overexpressed in Arabidopsis (Lü et al. 2013). Overexpressors of RhEXPA4 exhibit multiple modifications in their leaf blade epidermal structure which included smaller, compact cells and fewer stomata on leaves. It is likely that these modifications which are thought to be brought about by the action of expansins enabled the plants to be tolerant to the abiotic stresses such as drought and salt stress (Lü et al. 2013). Latest reports support the hypothesis that expansins are involved in conferring plant salt tolerance. It has been revealed that salt sensitive maize had reduced β-expansin protein while on the other hand, maintenance of the β-expansin protein is thought to have contributed to the better expansion capacity of the epidermal cell walls of the more resistant maize under salt stress (Zörb et al. 2015). This led to the conclusion that down regulation of the growth-mediating β-expansins reduced the expansion capacity of epidermal cells in the salt sensitive maize hybrid (Zörb et al. 2015).

Several other expansins have the ability to influence plant’s response to stress. When knocked down, *AtEXPA2* mutants showed a higher sensitivity to salt stress and osmotic stress while the opposite was true with the overexpressors of the same expansin gene (Yan et al. 2014). It is also thought that the increase in the level of *CpEXP1* observed in zucchini fruit under cold stress plays a significant role in improving chilling injury tolerance during postharvest cold storage in zucchini fruit (Carvajal et al. 2015). This concurs with Bauerfeind et al. (2015) who concluded that the expansin from their experiment appeared to act more as a counterbalancing agent against the growth-depressing effects of chilling exposure than as a mere growth promoter (Bauerfeind et al. 2015).

Although most studies support the notion that expansins enhances plant’s tolerance to abiotic stress, Kwon et al. (2008) opposed this notion. Yan et al. (2014) stated that overexpression of *AtEXPA2* gene enhanced salt tolerance and this was recently supported by Geilfus et al. (2015) who outlined that expansins have the ability to restore growth on growth reduced leaves under salt stress. However, after ectopically expressing some expansin genes, Kwon et al. (2008) reported that *AtEXP3* and *AtEXPB1* overexpressors became very sensitive to salt stress. There is therefore a need for further exploration of this area. Despite this, these researchers were in agreement with other researchers on the effects of expansins in enhancing growth and increasing leaf and petiole sizes through their effect on cell wall which enables plants to develop larger cells.

Expansins can also improve nutrient absorption from the soil. Studies have demonstrated that nutrient deficiency can stimulate the expression of expansins which will improve the plant’s root system and subsequently its ability to absorb nutrients such as phosphorus (P) from the soil even under low P levels (Guo et al. 2011; Li et al. 2014). This idea was recently endorsed by Zhou et al. (2014) when they overexpressed *GmEXPB2* in soybean and observed an increase in phosphorus efficiency. Li et al. (2015a) also concurred with this notion. They reported an increase in root network in overexpressors of the expansin gene *TaEXPB23* under the root-specific promoter PYK10. These transgenic plants showed an increased water uptake and performed better under drought probably because of the increased root to shoot ratio. All this is in line with the conclusion drawn by Li et al. (2014) who concurred with the idea that expansin proteins are involved in altered plant growth and development under nutrient stress conditions. They also postulated that the roles of expansins involved in this regard vary according to the nutrient and the particular expansin involved. This was after they observed that *GmEXPB2* was highly induced by phosphorus deficiencies treatment (Li et al. 2014), which is consistent with previous results (Guo et al. 2011) while several other different *GmEXPB*s also responded to different deficiencies including nitrogen, phosphorus, potassium and iron deficiencies (Li et al. 2014).

Even though there is a lot of evidence supporting the idea that expansins enhance plants’ tolerance to biotic and abiotic stress, there is need to validate this. This area is very important considering that a lot of the cultivated soils are becoming saline and less fertile while on the other hand heat stress and drought among other things are inevitable due to global warming and climate change. Inclusion of expansins such as *GmEXPB2* and *TaEXPB23* into breeding programs can enhance plants’ performance under nutrient limited conditions and drought conditions, respectively for example.

Plant diseases cause huge crop losses annually. It has been demonstrated that overexpression of the P450 gene CYP71Z2 in rice confers some resistance to the bacterial blight (Li et al. 2015b), which is partially contributed by the suppression of three rice α-expansin genes (*EXPA1*, *EXPA5* and *EXPA10*) and three rice β-expansin genes (*EXPB3*, *EXPB4* and *EXPB7*). This conclusion is in line with the notion which was propounded by Ding et al. (2008). They stated that suppression of expansion genes can prevent plant cell walls from loosening resulting in enhanced physical protection of plants against phytopathogens. This idea of suppressing expansins to enhance disease tolerance has been supported by Abuqamar et al. (2013) who reported an enhanced resistance to the necrotrophic fungi *Alternaria brassicicola* in *Atexpla2* mutant lines. This discovery is again an important starting point towards improved breeding for resistance to phytopathogens.

In areas where the parasitic weed striga (*Striga asiatica*) is present, it is a menace to farmers. However, before the plant-striga parasitism relationship is established, a haustorium must be formed. O’Malley and Lynn (2000) outlined that the process of haustorial organogenesis involves rapid arrest of root elongation, a redirection of cellular expansion from longitudinal to radial dimensions in the cells just distal to the root tip and the development and growth of haustorial hairs centrifugal to the swelling root tip. Since expansins are actively involved during cell expansion and haustorial development is critically dependent on cellular expansion, it is thought that expansins play a crucial role during this process. This has been confirmed by the identification of *SaExp1*, *SaExp2* and *SaExp3*, whose expression drastically increases during haustorium formation (O’Malley and Lynn 2000).

On the other hand, nematodes are obligatory biotrophic endoparasites which invade host roots and induce formation of syncytia, structures that serve them as the only source of nutrients. Just like haustorial organogenesis described above, syncytium development is characterized by extensive cell wall modifications (Fudali et al. 2008a, b). It is hypothesized that nematodes change expression of plant genes encoding cell wall modifying proteins including expansins (Fudali et al. 2008b). These researchers showed that two α-expansin genes (*LeEXPA4* and *LeEXPA5)* were up regulated in tomato roots infected with potato cyst nematode (*Globodera rostochiensis*). This is in-line with earlier studies (Ithal et al. 2007; Klink et al. 2007) in which microarray experiments revealed that expansin genes *EXPL2* and *EXPR3* were up-regulated in soybean roots infected with soybean cyst nematode (*Heterodera glycines*). Fudali et al. (2008b) also demonstrated that the cyst nematode development on transgenic plants carrying antisense construct of expansin was hampered which means that expansins can be a useful tool in crop improvement programs like breeding for resistance to nematodes.

Although expansins are universal in the plant kingdom they are also found in other organisms like snails where they are thought to have a degradative function in the digestive tracts (Cosgrove and Durachko 1994) and in a small set of phylogenetically diverse bacteria, fungi, and other organisms especially those that colonize plant surfaces (Georgelis et al. 2015). Several microbial expansin proteins have been discovered and reported. These include expansin-like proteins from the *Dictyostelium discoideum* (Kawata et al. 2015), *BsExlx1* from *Bacillus subtilis* (Kerff et al. 2008), *HcExlx2* from the marine bacteria *Hahella chejuensis* (Lee et al. 2010), *PcExl1* from the plant pathogenic bacteria *Pectobacterium carotovorum* (Olarte-Lozano et al. 2014) and *ScExlx1* from the Basidiomycete fungus *Schizophylum commune* (Tovar-Herrera et al. 2015). These microbial expansins have been shown to possess various capabilities which may be useful in enabling the microbes to attach and colonize plants. This is supported by the presence of such expansins in microbes such as plant pathogenic bacteria, including species of *Xanthomonas* and *Streptomyces* and fungal genomes which include plant pathogenic species of *Gibberella* and *Fusarium* (Georgelis et al. 2015) that colonize plants. In a broader sense, this presents an opportunity to plant breeders to breed for resistance to such pathogenic attack if the microbes rely solely on the action of their expansin genes. Also, some researchers are evaluating the potential use of these microbial expansins in cellulosic biomass conversion for biofuel production, as a means to disaggregate cellulosic structures (Georgelis et al. 2015).

### Future prospects

Even though not all expansins have the potential for application in crop improvement, several expansins including, but not limited to *SlEXPA6*, *LeExp1*, *RhEXPA4*, *TaEXPB23*, *GmEXPB2*, *OsEXPA2*, *OsEXPA17*, *PnEXPA1*, *GbEXPATR*, *MdEXPA12* and *NtEXPA4* and *5* have been proved to be useful for improving crops in various areas as highlighted in the text through overexpression and/or RNAi approaches for example. Taking tomatoes as an example, breeding for improved fruit shelf life is still a major objective. Expansins have been proved to play a pivotal role during fruit ripening and softening. Minoia et al. (2015) reported a massive 41 and 46 % fruit firmness enhancement in *Slexp1*-*6* and *Slexp1*-*7* mutant lines, respectively as compared to the control plants. This is in line with earlier reports by Brummell et al. (1999) who showed that suppression of *LeExp1* (another tomato expansin gene) inhibited polyuronide depolymerization and produced firmer fruits in transgenic tomatoes, while overexpression of the same gene resulted in softer fruits.

As such, new tomato varieties with enhanced fruit firmness could be generated by knock-out or suppression of *Slexp1* and *LeExp1* expansin genes using the new genome editing technologies especially Clustered Regularly Interspaced Short Palindromic Repeats (CRISPR) (Carroll 2014). As has been mentioned earlier on, this could be useful in breeding programs in combination with other alleles in the antioxidant or ethylene pathway since CRISPR/Cas can simultaneously introduce multiple gene disruptions (Wang et al. 2013) thus allowing breeders to edit multiple genes in one plant line through a single transformation (Xiong et al. 2015). Bearing in mind that previous manipulation of the polyamine or anthocyanin pathways had deleterious consequences on fruit quality traits such as flavor, texture and aroma despite their contribution in delaying fruit softening (Lovisetto et al. 2015), the employment of expansin could be a better option. Depending on the crop and objective, several other ways can be used to manipulate these genes.

## Conclusion

After observing the so many diverse roles played by expansins, it has been concluded that expansins are involved in many morphogenetic processes including germination, fruit ripening, growth of pollen tube, growth of root hairs, defoliation and many others which are yet to be discovered (Kuluev et al. 2013). Although there is still a need to further study and understand these expansins, especially considering that some expansins like *RhEXPA4* have been reported to negatively affect plant development when expressed at very high levels, it is also clear that incorporation of expansins in the crop improvement programs presents a potential tool to significantly improve crops in various aspects as highlighted in this paper. Although many crop traits are quantitative and are controlled by multiple genes, expansins, combined with other tools can be useful in manipulating many plant physiological aspects such as germination, stem development, yield and plant’s ability to withstand biotic and abiotic stress which has ever since become a concern following the current global warming and climate change issues. As highlighted in this paper, expansins can be used in floriculture industries to manipulate flower sizes through overexpression for example. Use of expansin together with other tools can enhance plants’ tolerance to abiotic and biotic stresses and can see a reduction in the use of chemicals or an improvement in the performance of plants under nutrient or salt stress while their use in fruit ripening manipulation can lower postharvest losses which currently hovers above 50 % for fruits and vegetables (Gustavsson et al. 2011). In short, the manipulation of expansins combined with other breeding tools can be a useful strategy to improve our crops. However, the worthy of this information lies in its utilization in crop improvement.

### **Author contribution statement**

Prince Marowa, Anming Ding and Yingzhen Kong drafted the manuscript. Prince Marowa collected background information. All authors read and approved the final manuscript.
